# Elevated Serum Uric Acid and Cardiovascular Disease: A Review and Potential Therapeutic Interventions

**DOI:** 10.7759/cureus.23582

**Published:** 2022-03-28

**Authors:** Michael Freilich, Andrew Arredondo, Seyedeh Leila Zonnoor, Isabel M McFarlane

**Affiliations:** 1 Internal Medicine, State University of New York Downstate Health Sciences University, Brooklyn, USA

**Keywords:** cardiovascular disease, hyperuricemia, hypertension, heart failure, gout disease

## Abstract

Several landmark studies found a relationship between elevated serum uric acid (SUA) levels and cardiovascular disease (CVD). In fact, the association between hyperuricemia and hypertension (HTN), coronary artery disease (CAD), and heart failure (HF) is currently well-established. While the mechanism linking hyperuricemia and CVD is not fully known, a systemic inflammatory response by the host is believed to play a role. With the goal of decreasing the morbidity and mortality of CVD in patients with hyperuricemia, the focus has now turned to properly optimizing a medication regimen for this patient population. Recent studies have shown that controlling underlying inflammation can, in fact, lead to better cardiovascular outcomes for populations with acute and chronic coronary disease. In this paper, we will discuss the current state of understanding on the association of hyperuricemia and cardiovascular disease. Furthermore, we will look into the most recent clinical trials showing the effects anti-inflammatory medications have on both decreasing and recovering from cardiovascular events. We will conclude with a discussion on, given the information mentioned above, how to properly optimize a medication regimen in patients with elevated SUA levels with a focus on decreasing the morbidity and mortality associated with CVD.

## Introduction and background

Introduction

Uric acid, a product of purine protein metabolism in humans, can be found in the blood and urine and in trace amounts in different organs of the body [1]. Hyperuricemia results from poor renal excretion or over-production of uric acid. Hyperuricemia can lead to gout, a systemic inflammatory process with uric acid crystals depositing in the intra-articular joints. Additionally, crystals can deposit in the renal tubules leading to uric acid stones, in the smooth muscle endothelium, and subcutaneously, termed tophi [2]. In the mid-1950s and 1960s, studies suggested a correlation between elevated levels of serum uric acid (SUA) and cardiovascular conditions. These studies highlighted a cardiovascular risk leading to a wide variety of pathologies, including hypertension (HTN), metabolic syndrome, coronary artery disease (CAD), vascular dementia, pre-eclampsia, and renal disease. Certain studies suggested that SUA levels do not need to be significantly elevated to pose a risk for the aforementioned cardiovascular conditions [3].

Other studies, however, argued that elevated SUA levels, regardless of extent, carried no risk factor burden, and had no correlation with the development of any cardiovascular disease (CVD). The Framingham Heart Study, renowned for its continuing research of over 70 years to identify risk factors specific for CVD, argued in the mid-1990s that elevated uric acid does not have a causal role in the development of CAD, death from CVD, or death from all causes, and that any apparent relationship is due to other confounding variables [4]. Expert researchers argued that there was no obvious or apparent mechanism between elevated SUA levels and the development of CVD. As time has evolved, the association between SUA levels and CVD gained further supporting evidence [5].

A 2008 NIH Public Access meta-analysis found a relationship between elevated uric acid and developing a cardiorenal disease, hypertension, metabolic syndrome, and diabetes. Furthermore, high levels of uric acid can predict obesity and chronic kidney disease [6]. To identify a relationship between high SUA levels and CVD, various studies have recently been conducted. One challenge noted is the simultaneous presence of established cardiovascular risk factors among individuals with elevated uric acid levels. It was argued that established modifiable and non-modifiable risk factors for cardiovascular disease (CVD), such as elevated blood pressure, smoking, diabetes mellitus, obesity, elevated cholesterol, age, gender, and genetics, could not be sufficiently controlled, making the high SUA levels as an independent risk factor for CVD difficult to establish. Another obstacle was identifying a clear pathogenetic mechanism for SUA levels to cause CVD [6]. Both of these challenges will be discussed in this review.

Background

Uric Acid Biochemistry

Uric acid is a product of purine protein metabolism and is usually found as a monosodium salt under normal physiologic conditions with normal blood pH of 7.35-7.45. The more predominant form is nonionized uric acid under more acidic conditions such as in the urine. Serum uric acid levels result from several significant interactions: dietary purine intake, the extent of purine metabolism, and rate of urinary and gastrointestinal (GI) excretion/breakdown. When absorbed through the GI tract, xanthine oxidase converts oxy-purines, which are found in the diet, to uric acid. Xanthine oxidase is found in the gut mucosa of the small intestine, as well as in the liver. Hyperuricemia is defined as elevated SUA levels that exceed 6.0 mg/ml in females and 7.0 mg/ml in men [7]. This is based on reductive and oxidative laboratory techniques that are widely utilized to quantify uric acid serum levels based on wavelength and supersaturation, respectively [7]. Epidemiology

Epidemiology

Cardiovascular disease: According to the American Heart Association, around one out of every four American deaths is related to CVD [8]. Furthermore, mortality from CVD is the number one cause of international mortality. According to the World Health Organization, around 17.7 million deaths in 2015 were attributed to some form of cardiovascular disease. Among the spectrum of diseases found in cardiovascular pathology, CAD is the leading cause of mortality. CAD risk factors include smoking, HTN, diabetes mellitus, elevated cholesterol and lipids, poor nutrition, and obesity. The risk of acquiring CVD remains high, with a substantial 50% risk by age 45 in the general population [9]. CVD provides an enormous economic burden on the overall healthcare system and is expected to cost $368 billion by 2035 [9].

Gout: Gout is a highly prevalent disorder that affects up to 6.8% of the population [10]. As mentioned above, hyperuricemia leads to crystal deposition secondary to the supersaturation of uric acid at physiological pH (7.35-7.45). It deposits in various tissues, forming most commonly in musculoskeletal joints, subsequently leading to inflammatory arthritis. Gout is one of the most common causes of inflammatory arthritis in men and can be episodic, with recurrent exacerbations of painful symptoms, known as flares. Along with musculoskeletal joints, these monosodium urate crystal deposits can also manifest in the kidneys, leading to uric acid stones and soft tissue deposition with tophi development. Gout has been associated with both modifiable and non-modifiable risk factors, which are summarized below (Table 1) [11].

**Table 1:** Risk factors for the development of gout

Modifiable Risk Factors
Dietary (Increased purine metabolites and turnover, Alcohol, Red meats, Seafood, Fructose)
Obesity
High Blood Pressure
Diabetes Mellitus / Elevated Serum Glucose (Increased insulin resistance can decrease renal excretion)
Environmental Lead (low doses)
Non-Modifiable Risk Factors
Age
Gender (Male > Female)
Genetics - Congenital errors of metabolism, Lesch-Nyhan (deficiency of the enzyme hypoxanthine-guanine phosphoribosyltransferase), Phosphoribosyl pyrophosphatase synthetase-related disease, Excessive cell death/ generation, Glycogen storage diseases
Other Etiologies
Medication-induced Diuretics, Thiazides, Antiplateles, Beta-blockers
Osteoarthritis
Chronic Kidney Disease

## Review

Mechanism of action for hyperuricemia and CVD

The mechanism of CVD following hyperuricemia has yet to be fully proven; however, some hypotheses may explain the link between the two. The hyperuricemia associated with gout causes a systemic inflammatory reaction that can affect various organs in the body. This can be thought to occur as inflammatory markers, such as erythrocyte sedimentation rate (ESR) and C-reactive protein (CRP), are increased during an acute gouty attack, as found in a study conducted at the Boston Veterans Administration Medical Center. The same study also showed some correlation between the rise in inflammatory markers and the number of joints involved in the attack [12]. The development of atherosclerosis involves an inflammatory reaction by the host and when combined with a systemic underlying inflammatory disorder, this can be thought to exacerbate the progression of cardiovascular disease. Other inflammatory diseases, such as systemic lupus erythematosus and rheumatoid arthritis, have also been shown to increase the risk of CVD, giving credence to this potential mechanism of why patients with hyperuricemia have an increased risk of developing CVD [13-14].

Other proposed mechanisms as to why hyperuricemia can lead to CVD include the relationship between hyperuricemia and endothelial dysfunction. Uric acid is thought to increase the inflammatory cytokine of high-mobility-group-box chromosomal protein-1 (HMGB1), leading to endothelial dysfunction through oxidative stress and inflammation. A study published in BioMed Research International found increasing levels of uric acid decrease the amount of nitric acid released from endothelial cells, leading to endothelial dysfunction. This increases the risk of atherosclerotic plaque buildup, leading to CV disease [15]. The final mechanism discussed here incorporates the prolonged immobility gout patients have, secondary to the amount of pain they are in. This can alter the cardiovascular system through decreased cardiac muscle mass, leading to decreased stroke volume. This leads to a responsive increase in heart rate, which can ultimately lead to various CV diseases. Immobility also leads to a prothrombotic state, which can cause many forms of CV disease, stroke, and myocardial infarction [16].

Hyperuricemia and HTN

There is substantial experimental and clinical evidence that supports the hypothesis that elevated SUA levels may lead to HTN. Independent from other risk factors, numerous research studies have shown that hyperuricemia within five years increases the relative risk for HTN [6]. Elevated SUA levels were observed in 25-60% of patients with untreated HTN and nearly 90% of adolescents with recent-onset essential HTN. These studies have also shown that the positive correlation between elevated SUA levels and HTN decreases with increasing age and onset of HTN. This suggests that the association between SUA levels and HTN is more significant in younger patients with early-onset HTN [6].

The first animal studies have shown that increased SUA levels, whether mild or moderate, led to increases in blood pressure. Later in 2001, a new experimental rats study confirmed the positive correlation between uric acid and the development of HTN. Since rats have active uricase in their blood serum, an irreversible competitive inhibitor was given to induce hyperuricemia. After reaching elevated levels of SUA, several weeks later, a corresponding increase of blood pressure was recorded with a 10 mmHg increase for each 0.5 mg/dL increase of SUA levels. Furthermore, the introduction of a xanthine oxidase inhibitor or a uricosuric medication, both of which would lower SUA levels, ceased the development of hypertension in this group [17].

One proposed mechanism explaining this was shown to result from renal vasoconstriction in response to elevated SUA levels, which resulted in a significant decrease in endothelial nitric oxide (NO). Other experimental rat studies have shown that uric acid can induce cellular proliferation, initiate an inflammatory response, create oxidative stress, and increase the renin-angiotensin pathway's activity [6]. Endothelial dysfunction and increases in plasma renin activity due to a systemic inflammatory reaction can ultimately lead to hypertension, as was noted in the studies above.

Clinical data: allopurinol

There is evidence that histologic changes of the renal microvasculature due to hyperuricemia resemble that of long-standing arteriosclerotic changes. Allopurinol, a xanthine oxidase inhibitor, has been shown to help lower blood pressure in some studies. Feig et al. recruited 30 adolescents with ages ranging from 11 to 17 years who developed newly diagnosed hypertension with coexisting serum uric acid levels greater than or equal to 6 mg/dL. Exclusion criteria include those with long-standing hypertension or known renal, cardiovascular, GI, or endocrine diseases. Allopurinol administration leads to decreases in the mean systolic blood pressure of 6.9 mmHg (95% CI, -4.5 to-9.3 mmHg) and -5.1 mmHg for diastolic pressure (95% CI, -2.5 to-7.8 mmHg). Though the sample size was limited, it showed promising developments of allopurinol use as an adjunct to help manage new-onset hypertension [18].

In a 2014 retrospective cohort study by Beattie et al., patients with hypertension, of age older than 65, were prescribed allopurinol with pretreatment, and BP readings were followed during treatment. Data from similar demographic patients not on allopurinol were obtained and represented controls. Primary outcomes were changes in the blood pressure of patients on stable blood pressure medication. In total, 365 patients received allopurinol and were compared to 6678 controls. Allopurinol use was found to be independently associated with both a decrease in systolic and diastolic blood pressure, with a more significant reduction in the high-dose allopurinol group. However, the change in blood pressure was not related to baseline uric acid level. This study is notable, as it shows a relationship between lowering SUA levels with lowering blood pressure, giving more credence to the belief that elevated SUA levels are associated with the development of HTN [19].

Clinical data: febuxostat

Febuxostat is another medication that can be used to treat hyperuricemia or gout. Febuxostat is a non-purine analog xanthine oxidase inhibitor that ultimately decreases the amount of uric acid produced. Febuxostat, like allopurinol, has been shown to decrease systolic blood pressure in patients with hyperuricemia with concomitant hypertension and normal renal function. A randomized study consisting of 121 subjects with simultaneous hyperuricemia and hypertension was divided into two groups, with one receiving 80 mg febuxostat daily and the other obtaining a placebo pill. A statistically significant drop in both systolic and diastolic blood pressure was found in patients in the treatment group, of 6.6 mm Hg and 3.3 mm Hg, respectively. These results were seen after treatment for six weeks. Febuxostat also showed a statistically significant drop in SUA levels over this time [20]. The CARES (Cardiovascular Safety of Febuxostat and Allopurinol in Participants With Gout and Cardiovascular Comorbidities) trial compared allopurinol to febuxostat in patients with both gout and cardiovascular disease. It concluded that while febuxostat use was considered “non-inferior to allopurinol with respect to rates of adverse cardiovascular events,” both CV and all-cause mortality were higher in the febuxostat group [21]. The CARES trial led to the Food and Drug Administration (FDA) releasing a warning on a potential connection between febuxostat use and CVD. However, most recently, the FAST trial dispelled this notion and showed febuxostat use, when compared to allopurinol, showed no increase in a major CV event or all-cause mortality [22].

Hyperuricemia and heart failure

More recently, increased focus has been placed on the relationship between hyperuricemia and heart failure (HF). In a recent study, 5,713 patients without a history of HF, congenital heart defects, or stroke between 2003-2007 were analyzed. The mean age was 65.5, and hospitalizations due to heart failure, strokes, and all-cause mortality were monitored over a median follow-up period of 10 years duration. With regards to HF hospitalizations, the incidence rates for patients with and without gout were 13.1 and 4.4, respectively, per 1000 person-years. Of the population studied, 3.3% had gout, and these patients were older, heavier, had lower educational experience, and had lower total and HDL cholesterol. Multivariable adjustments showed a hazard ratio (HR) of 1.97. The study further showed an increased risk of HF with both preserved and reduced ejection fraction in patients with elevated SUA levels. The researchers concluded that gout is a risk factor for the development of heart failure in adults of this age group [23].

A second study aiming to find the relationship between hyperuricemia and HF studied 4,989 patients with a mean age of 36 who had no evidence of heart failure at the beginning of the study. It was found that patients with gout had a two to three times increased risk of heart failure compared to patients without gout, with an HR of 1.74. Furthermore, systolic dysfunction confirmed by echocardiogram measures were increased in this patient population as well, as measured by left ventricular ejection fraction and global left ventricular systolic dysfunction. Mortality was seen to be elevated in participants with gout and heart failure, with an adjusted HR of 1.5. This study concluded gout increased one's risk for being diagnosed with HF, a similar finding to the paper mentioned in the paragraph above [24].

While the studies above concluded elevated SUA levels were a risk factor for the development of heart failure, they failed to isolate the relationship between these two, as patients in these studies had other cardiovascular risk factors. Therefore, it was difficult to tell if elevated SUA levels alone led to an increased risk of HF or if other conditions contributed to the development of HF. It is for this reason a study was conducted in the Kangjian Community Health Center of Shanghai where researchers tried to directly answer this question. In this study, patients with “hypertension, diabetes mellitus, preexisting cardiovascular disease, hyperlipidemia, overweight or obesity, a history of gout or hyperuricemia and were taking medication for their condition, or chronic kidney disease” were excluded [24-25]. It followed 2,749 patients with the above criteria over the age of 65 over a four-year period. In this population, 6.5% of patients with hyperuricemia developed a CHF event while 3.1% of patients without hyperuricemia developed a CHF event. The conclusion of this study was patients with asymptomatic hyperuricemia had a 2.34 increased risk of developing a CHF event [25]. Another study obtained using data from MJ Health Screening Centers in Taiwan also isolated elevated SUA levels and the development and mortality of CHF. It found uric acid levels over 7 mg/dL increased mortality from CHF, which “increased 13% for every 1 mg/dL increase” in SUA levels. This study, like the one listed above it, concluded that elevated SUA levels were an “independent risk factor of mortality from all causes, total CVD, and ischemic stroke in the Taiwanese general population, in high-risk groups, and potentially in low-risk groups” [26].

Hyperuricemia and CV outcomes in patients with obstructive CAD

The next association that will be examined is the relationship between elevated SUA levels and CV outcomes in patients with CAD. A new clinical trial study enrolled 85,173 patients who had undergone cardiac catheterization showing obstructive CAD. Of this group, 1,406 had gout, and patients were monitored for cardiac events and all-cause mortality over a median follow-up period of 6.4 years. This was an important study because while the relationship between gout and CV outcomes had been studied before, very few focused on this particular group of patients who had baseline CAD. The study concluded that the patients with gout and baseline CAD had an increased rate of all-cause mortality [27].

Furthermore, “postbaseline gout diagnosis was associated with a >2‐fold increase in the risk of heart failure death” [27]. These patients were also found to be 40% more likely to reach the prior composite endpoint of CV death, myocardial infarction (MI), or stroke compared to the group without gout. This reiterated the results of a prior study of over 50,000 males in the Health Professionals Follow-Up Study. These results found that patients with gout and coronary artery disease had a 26% increased risk of mortality due to an underlying cardiac cause. The increased risk of CV death was notable in this subset of patients, as many were on a medication regimen meant to target and mitigate CVD mortality. This may imply that there is additional risk in these patients that these medications do not cover, opening a discussion on how to better medically optimize this group [28].

Another study, the Coronary Disease Cohort Study, looked at 1,514 patients who initially presented with an acute coronary syndrome. This study found no rise in mortality rate in patients with gout but did conclude “time to readmission for heart failure was significantly briefer in those with, compared to those without gout.” It also found that increasing serum uric acid levels were associated with a higher chance of death and hospital readmission “for either a cardiovascular event or heart failure.” This study also found that in these patients who had an ACS event, the risk of readmission for HF and longer hospital stays were seen to move in unison with elevated SUA levels [29]. These three studies are important because while the relationship between gout and CV outcomes had been studied before, very few focused on this particular group of patients who had baseline CAD.

Discussion

As seen above, there is thought to be a relationship between elevated SUA levels and the development of CVD. The potential mechanism explaining the link between the two is thought to be related to the underlying inflammation or increase in IL-1 seen in flares of gout. IL-1 is an inflammatory cytokine and is believed to play a role in the progression of heart failure with preserved ejection fraction, as shown in multiple animal models [30-31]. It is, for this reason, researchers have recently started to look at the relationship between decreasing inflammation and its impact on CVD development and recovery. The Canakinumab Anti-Inflammatory Thrombosis Outcomes Study (CANTOS) clinical trial is a randomized, double-blind study that recruited 10,061 patients with a previous diagnosis of myocardial infarction, along with elevated C-reactive protein levels of 2 mg/l. Patients were either given canakinumab, a monoclonal antibody targeting interleukin (IL)-1, or a placebo. If patients were given canakinumab, they were given one of three doses and were followed over a four-year period with the primary outcomes of non-fatal myocardial infarction, stroke, or death due to cardiovascular causes measured. The study showed a decreased risk of HF hospitalization in patients with a previous myocardial infarction and elevated c-reactive protein (CRP) on anti-IL-1 therapy. Over the four-year period, it was discovered that patients on moderate-dose therapy of canakinumab (150 mg every 3 months) led to a significantly lower incidence of recurrent cardiovascular events, such as MI and stroke, when compared to placebo (HR: 0.83; 95% CI 0.73-0.97 P=0.005). One can infer from this that targeting inflammation in an already elevated state can “significantly lower rate of recurrent cardiovascular events than placebo, independent of lipid-level lowering” [32].

After the results of the CANTOS trial were published, further investigation looked at this relationship between anti-inflammatory medications and CV outcomes. The COLCOT (Colchicine Cardiovascular Outcomes Trial) is a randomized, double-blind trial that looked at 4,745 patients within 30 days of an MI. Patients either received 0.5 mg colchicine once per day or a placebo. Colchicine counteracts inflammation by preventing microtubule formation, “microtubule-based inflammatory cell chemotaxis, generation of leukotrienes and cytokines, and phagocytosis” [33]. After following patients for a median follow-up period of 22 months, the patients receiving colchicine had a lower rate of “death from cardiovascular causes, resuscitated cardiac arrest, myocardial infarction, stroke, or urgent hospitalization for angina leading to coronary revascularization.” The percentage of patients in the colchicine and placebo groups who experienced these outcomes were 5.5% and 7.1%, respectively. The major difference between these two groups was seen in the reduction in strokes and urgent revascularizations in the patients taking the colchicine. Other notable differences between the groups were an increased incidence of diarrhea, nausea, infection, and pneumonia in the group taking colchicine. In conclusion, colchicine was seen to benefit this group of patients, thought to be due to its anti-inflammatory properties [34]. It is important to note that almost all of the patients in both groups were also taking aspirin, another antiplatelet medication, and some sort of statin. This is important because these patients were medically optimized from a cardiac perspective and the addition of an anti-inflammatory drug to a classic cardiac medication regimen decreased CVD morbidity and mortality in the patient population listed above.

Shortly after the results of the COLCOT trial were published, the LoDoCo2 (Low-Dose Colchicine-2) trial focused on colchicine and its impact on people with chronic coronary disease, something the prior study did not do. In this study, 5,552 patients were looked at with some receiving 0.5 mg colchicine daily and the others receiving a placebo. Similar to the results of the CALCOT study, 6.8% of patients in the colchicine group experienced the primary end-point event compared to 9.6% of the control group not experiencing it. The study confirmed that the incidence rates of spontaneous myocardial infarction or ischemia-driven coronary revascularization (composite end-point), cardiovascular death or spontaneous myocardial infarction (composite end-point), ischemia-driven coronary revascularization, and spontaneous myocardial infarction were also significantly lower with colchicine than with placebo.” This allowed the study to conclude that 0.5 mg of colchicine given once per day was beneficial to patients with chronic CAD, as it significantly reduced the risk of having a CV event [35].

The CANTOS, COLCOT, and LoDoCo2 trials showed a decrease in CV events or recovery times when given anti-inflammatory medication. As mentioned above, the link between hyperuricemia and CVD development is thought to be related to some sort of systemic inflammatory reaction. While these trials did not directly look at patients with hyperuricemia and its relative effect on CV outcomes, one may infer that these anti-inflammatory medications may have the same impact on this patient population as it does on the general population studied in these trials. It is, for this reason, we would like to open this discussion and possible future research into examining the efficacy of anti-inflammatory medications' impact on CV outcomes in patients with elevated SUA levels [33-35].

## Conclusions

After extensive review, recent research shows a relationship between the effects of elevated SUA levels and its role as an independent risk factor for developing subsequent CVD. Multiple mechanisms have been proposed linking the two with many related to the underlying systemic inflammation elevated SUA levels cause. Focus has recently turned to the impact of anti-inflammatory medications and their role in decreasing morbidity and mortality with regards to CVD. While these studies never directly studied these particular treatments in patients with elevated SUA levels, one may be able to infer that these patients may benefit from medications in this class. We have outlined the research above and want the discussion to focus specifically on patients with elevated SUA levels and for them to be treated with anti-inflammatory medications. We would like to see how this group responds to this regimen with a focus on its potential decreased incidence of morbidity and mortality related to CVD. Larger sample sizes and more effective time periods are needed to truly identify the effects of these therapies long-term.
